# Identifying Co‐Expressed lncRNAs Correlated With Traits of Interest in an Animal Model for Metabolic Diseases in Humans

**DOI:** 10.1002/age.70201

**Published:** 2026-09-08

**Authors:** Lucas Echevarria Nascimento, Bruna Pereira Martins da Silva, Camila Sabino de Oliveira, Mariah Castro Durval, Felipe André Oliveira Freitas, Fernanda Nery Ciconello, Izally Carvalho Gervásio, Simara Larissa Fanalli, Pablo Augusto de Souza Fonseca, Beatriz Gutiérrez‐Gil, Vivian Vezzoni de Almeida, Aline Silva Mello Cesar

**Affiliations:** ^1^ Department of Animal Science, Luiz de Queiroz College of Agriculture University of São Paulo Piracicaba Brazil; ^2^ Department of Animal Sciences Purdue University West Lafayette Indiana USA; ^3^ Department of Veterinary Medicine, Faculty of Animal Science and Food Engineering University of São Paulo Pirassununga Brazil; ^4^ Instituto de Ganadería de Montaña (CSIC‐Univ. de León) León Spain; ^5^ Department of Animal Production Universidad de León León Spain; ^6^ Department of Animal Science, School of Veterinary Medicine and Animal Science Federal University of Goiás Goiânia Brazil

**Keywords:** disease, fatty acids, pig model, regulatory RNAs, WGCNA

## Abstract

Nutrigenomics investigates how nutrients modulate gene expression. Among them, fatty acids (FA) play important roles in regulating gene transcription, while long non‐coding RNAs (lncRNAs) may be associated with gene regulation and metabolic diseases. This study aimed to analyze the hepatic transcriptome of pigs, a species frequently used as a model for nutrigenomic studies, to identify novel lncRNAs and their potential target genes in response to diets containing different sources of FA. Seventy‐two pigs were fed four diets supplemented with 1.5% soybean oil (control), 3% canola oil, 3% fish oil, and 3% soybean oil. RNA sequencing of liver samples was performed to identify novel lncRNAs. Weighted Gene Co‐expression Network Analysis (WGCNA) was used to identify modules associated with phenotypic traits related to lipid metabolism and inflammation. Functional enrichment analyses were then conducted to annotate genes within these modules using Gene Ontology (GO) terms and to assess overlap with Quantitative Trait Loci (QTL). The results revealed 106 novel lncRNAs potentially regulating genes associated with lipid metabolism and immune responses in pigs fed diets with different FA sources. These findings enhance understanding of the regulatory role of lncRNAs in pigs and reinforce their relevance as models for human metabolic diseases.

## 1 Introduction

Fatty acids (FA) play an important role in the constitution of the cell membranes, especially in the phospholipid bilayer (Nagy and Tiuca 2017; Sayanova and Napier 2004). Phosphatidylcholine is the most abundant class of phospholipids and plays an essential role in maintaining the integrity and functionality of biomembranes. It serves as a precursor for other polar lipids, participates in cell signaling processes, and influences lipoprotein concentration (Ali and Szabó 2023). Additionally, the ratio of FA in cellular membranes reflects universal cellular signaling mechanisms and inflammatory responses (Ali and Szabó 2023). Fatty acids can be classified into three types: saturated (SFA), polyunsaturated (PUFA), and monounsaturated (MUFA) (Zárate et al. 2017). Linoleic acid (LA, C18:2 n‐6) and oleic acid (OA, C18:1 n‐9) are found primarily in oils, such as soybean and canola oil, while alpha‐linolenic acid (ALA, C18:3 n‐3) is found in flaxseed oil and walnuts. Eicosapentaenoic acid (EPA, C20:5 n‐3) and docosahexaenoic acid (DHA, C22:6 n‐3) are also commonly found in fish oil (Fanalli, da Silva, Gomes, et al. 2022; Fanalli, da Silva, Petry, et al. 2022).

Studies carried out with animals, including mice and humans, have reported that the ideal intake of omega‐6 (n‐6) and omega‐3 (n‐3) can prevent overweight and the risk of developing obesity. While high dietary intake of n‐6 and SFA may increase leptin and insulin resistance, n‐3 may aid in homeostasis and weight loss in humans (Risérus 2008; Simopoulos 2016). Fatty acids have the function of modulating the release of different cytokines (Meydani 1992). Highly unsaturated FA, such as PUFA, n‐3, have gained significant attention for its potential anti‐inflammatory properties (Meydani 1992). Wei et al. (2010) showed that stable cell production through n‐3 could increase insulin secretion and confer strong resistance to cytokine‐induced β‐cell destruction.

Pigs are closer to humans than mice in protein structural similarity and at the genetic level (Dawson et al. 2017). Organs, physiology, and progression of metabolic and infectious diseases are also similar between pigs and humans; thus, the study of human diseases and their understanding can be enhanced due to the knowledge generated by the genetic and physiology studies in pigs. Hence, the pig is an animal model for several areas of research, and the study of different pathologies in this species can consequently provide a better understanding of human diseases (Bassols et al. 2014; Lunney 2007).

Consumption of FA is involved in metabolic pathways and can be associated with health and disease. Oleic acid, LA, DHA, and EPA can regulate gene transcription in various tissues and organs, such as skeletal muscle, adipose tissue, the liver, and the brain (Fanalli, da Silva, Gomes, et al. 2022; Fanalli, da Silva, Petry, et al. 2022). Liver plays an important role in physiological and metabolic studies (Nguyen et al. 2008) and in the innate immune response through lymphocytic cells, including natural killer (NK) and natural killer T (NKT) cells (Gao et al. 2008; Li and Diehl 2003).

Methodologies using transcriptome data highlight the potential of RNA‐seq to study global expression profiles in tissues of interest. In pigs, for example, RNA‐seq has been applied not only to characterize overall gene expression but also to investigate long non‐coding RNAs (lncRNAs), some of which have been shown to play regulatory roles in transcriptional regulation. In a recent study conducted by our group, RNA‐seq was applied to demonstrate that different types of oils, such as canola oil and fish oil, modulate the expression of mRNA involved in metabolic pathways in the liver (Fanalli et al. 2023).

Studies analyzing lncRNAs in pigs are still lacking in the field of nutrigenomics. Some aspect of interest is their tissue‐specific activity, as lncRNAs are involved in transcriptional regulation, cell differentiation, signaling, and epigenetic processes (Kumar et al. 2019). lncRNAs are currently being used to understand the health and economic traits of interest in various animals, as well as diseases in humans (Yoon‐Been and Jun‐Mo 2023). Single‐nucleotide polymorphisms (SNPs) related to FA have already been identified in pigs involved in processes such as desaturation and the Wnt/β‐catenin pathway (Liu et al. 2024). More accurate analyses of lncRNA can contribute to finding possible means to develop the diagnosis, treatment, and prevention of diseases (Sun et al. 2020).

In this context, this study aimed to analyze the pig liver transcriptome to identify novel lncRNAs and their potential target genes modulated by the consumption of different sources of dietary FA through the analysis of co‐expression networks.

## 2 Results

### 2.1 Sequencing, Identification of Novel lncRNAs, and Selection of Genes for Analysis of Co‐Expression and Correlation Networks

During RNA‐seq analysis, one of the samples from the 1.5% Soybean Oil (SOY1.5) treatment did not pass quality control, resulting in a total of 71 RNA samples that were sequenced. NanoDrop‐generated concentration data and RNA Integrity Number (RIN) values are available in Data S1.

A total of 25 941 genes were identified (expression values equal to or greater than 0) after the quantification analysis. Of these, 124 novel lncRNAs were identified. Filters were applied for each treatment (FPKM > 0.2 for at least 50% of the samples), and the total number of annotated genes, including lncRNAs and protein‐coding genes, is shown in Table 1. More detailed information on the novel lncRNAs identified in this study, such as genome location and sequences, is available in Data S2.

**TABLE 1:** Total number of genes identified for each group of treatment samples, including novel lncRNAs and protein‐coding transcripts after filtering. Samples with FPKM expression values > 0.2 for at least 50% of the samples were retained.

Treatment	Genes	Novel lncRNAs	Protein coding
SOY1.5	12.881	110	12.588
SOY3.0	13.085	110	12.662
FO	12.925	108	12.628
CO	12.944	111	12.647

*Note:* Samples with FPKM expression values > 0.2 for at least 50% of the samples were retained.

Abbreviations: Genes, total number of genes identified after the filters applied; Novel lncRNA, amount of novel lncRNA identified per treatment; Protein coding, total protein‐coding genes found; Treatment, Dietary treatment used.

**FIGURE 1:**
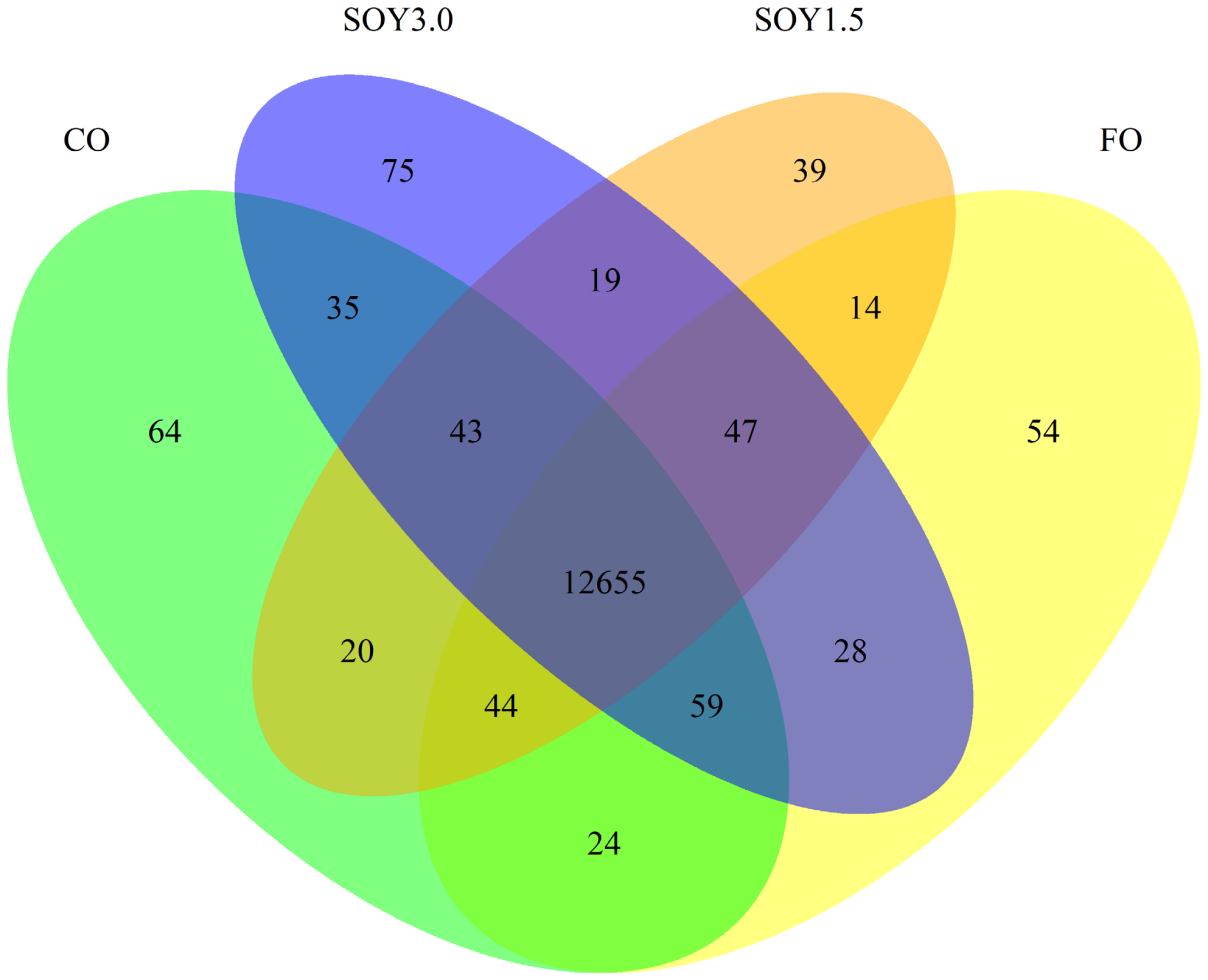
Visualization of unique and shared genes among all the dietary treatments considered in this work, including novel lncRNAs and coding proteins, through the Venn diagram.

Figure 1 shows the Venn diagram of the comparisons regarding the genes quantified in each treatment (CO: Canola Oil; FO: Fish Oil; SOY3.0%: 3.0% Soybean Oil), where SOY1.5 vs. FO, SOY1.5 vs. CO, SOY1.5 vs. SOY3.0, FO vs. CO, FO vs. SOY3.0, and CO vs. SOY3.0 presented 14, 20, 19, 24, 28, and 35 shared genes, respectively. Unique genes were also found for each diet as follows: CO (64 unique genes), SOY1.5 (39), SOY3.0 (75), and FO (54). Among all the dietary treatments, 12 655 genes were identified in common, of which 106 were novel lncRNAs. These 12 655 genes were used for the WGCNA. The unique genes for each type of comparison between treatments and their classifications are provided in Table S1.

### 2.2 Co‐Expression Analysis of Genes Shared Between Dietary Treatments

In total, 73, 46, 51, and 73 modules were identified for the SOY1.5, SOY3.0, FO, and CO treatments, respectively. Four modules were identified as unique to CO in relation to SOY1.5, being *darkslateblue* (198 genes), *orangered3* (112 genes), *saddlebrown* (111 genes), and *skyblue1* (213 genes). The FO treatment showed two unique modules in relation to SOY1.5, *floralwhite* (266 genes) and *saddlebrown* (239 genes).

Unique modules were not found for the SOY3.0 treatment compared to SOY1.5. Comparisons between dietary treatments, number of genes, and lncRNA found within each unique module are shown in Table 2, while the total list of modules (unique, shared, significant, and non‐significant) and their genes can be seen in Table S2.

**TABLE 2:** Number of hub genes and novel lncRNAs present in comparison with 1.5% soybean oil (SOY1.5) for each treatment that presented modules considered as unique.

Treatment	Module	Genes	lncRNAs
CO	*darkslateblue*	198	2 novel
CO	*orangered3*	112	—
CO	*saddlebrown*	111	2 novel
CO	*skyblue1*	213	—
FO	*floralwhite*	266	6 novel
FO	*saddlebrown*	239	1 novel

Abbreviations: Genes, total number of genes grouped within each module; lncRNAs, amount of novel lncRNAs; Module, modules identified as unique; Treatment, Dietary treatment used.

### 2.3 Correlation of Unique Modules With Phenotypes of Interest

Correlations between the identified unique modules and phenotypes of interest were tested, and significant modules (*p*‐value < 0.05) were selected. The *darkslateblue* module, which was unique for CO, had significant correlations (*p*‐value < 0.05) with four of the phenotypes evaluated, SFA (−0.48, *p*‐value 0.04), Monosaturated fatty acid (MUFA) (0.54, *p*‐value 0.02), Tumor necrosis factor (TNF)‐α (0.67, *p*‐value 0.05) and Interleukin (IL)‐6 (−0.68, *p*‐value 0.04); the *orangered3* module also showed correlation with Interleukin (IL)‐6 (−0.73, *p*‐value 0.02), whereas *saddlebrown*, also from the CO treatment, correlated only with Ether extract of muscle (EEM) (−0.59, *p*‐value 0.009). The *skyblue1* module showed no correlation with any of the phenotypes evaluated in this study.

The FO treatment showed significant correlations for three phenotypes in only one of its unique modules, *floralwhite*, being correlated with SFA (−0.54, *p*‐value 0.02), MUFA (0.50, *p*‐value 0.03), and Interleukin (IL)‐18 (0.69, *p*‐value 0.04). A potential target gene in cis was detected, the *FGF11* gene, as a possible target for the lncRNA MSTRG.75, present in the *floralwhite* module of the FO treatment. The other unique modules used for this analysis, *darkslateblue* and *saddlebrown* (both from CO), did not present genes in cis according to the parameters used. The FO *saddlebrown* module did not show any correlation with phenotypes. These correlation estimates are provided in Table 3, while data on correlations between all modules classified as shared, significantly (*p*‐value < 0.05) for each treatment, are available in Table S3.

**TABLE 3:** Modules significantly correlated with phenotypes of interest for the dietary treatments that presented unique modules in comparison with the reference treatment based on 1.5% soybean oil (SOY1.5).

Treatment	Module	Phenotype	Correlation	*p*
CO	*saddlebrown*	EEM	−0.59	0.009
CO	*darkslateblue*	MUFA	0.54	0.02
CO	*orangered3*	IL‐6	−0.73	0.02
CO	*darkslateblue*	SFA	−0.48	0.04
CO	*darkslateblue*	IL‐6	−0.68	0.04
CO	*darkslateblue*	TNF‐α	0.67	0.05
CO	*skyblue1*	—	—	—
FO	*floralwhite*	SFA	−0.54	0.02
FO	*floralwhite*	MUFA	0.5	0.03
FO	*floralwhite*	IL‐18	0.64	0.04
FO	*saddlebrown*	—	—	—

Abbreviations: Correlation, correlation value for the phenotype (between −1 and 1); Module, unique module referring to treatment; *p*‐value, significance value of the correlation (*p*‐value < 0.05); Phenotype, phenotype correlated with the modulus; Treatment, Dietary treatment used.

**FIGURE 2:**
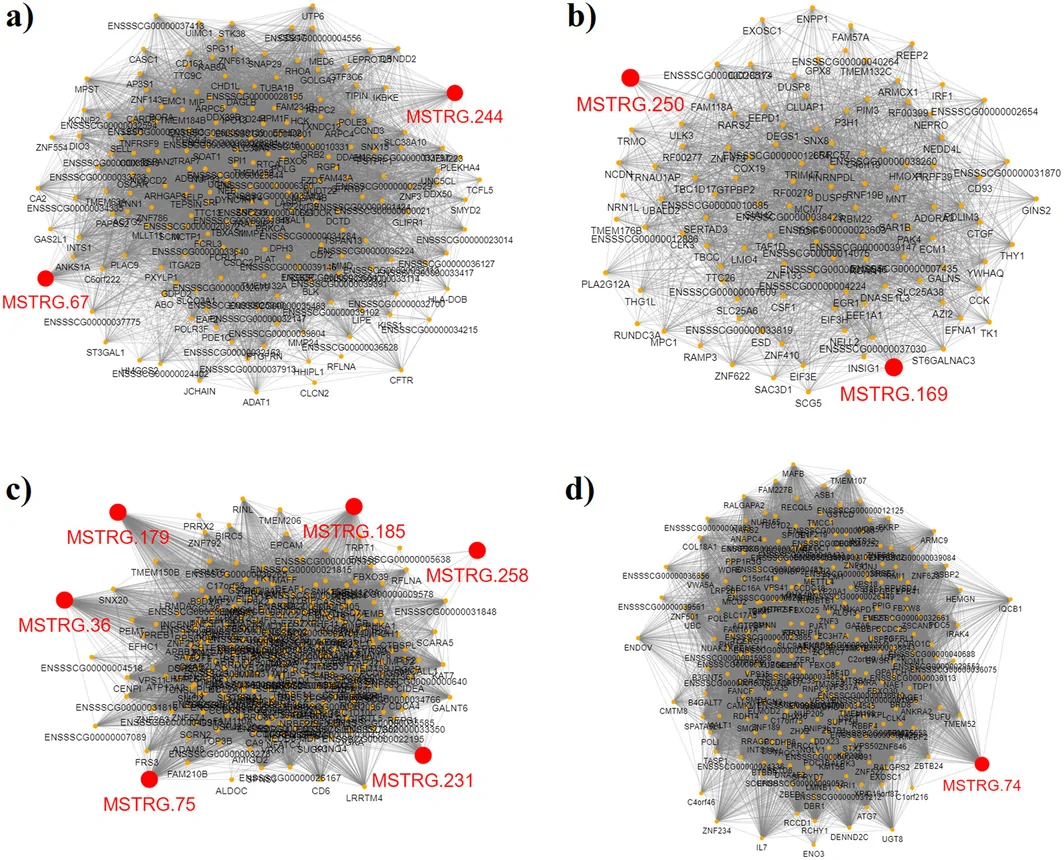
lncRNAs co‐expression networks for genes in unique modules of each treatment that presented at least one novel lncRNA: Canola: (a) *Darkslateblue* (2 novel); (b) *Saddlebrown* (2 novel); Fish: (c) *Floralwhite* (6 novel), and (d) *Saddlebrown* (1 novel). The novel lncRNAs are highlighted in red, while the linked genes are highlighted orange.

To identify the possible targets of the lncRNAs identified in the present study, especially the new ones, an interaction network between the lncRNAs and other genes for each unique module of each treatment that presented at least one novel lncRNA was generated (Figure 2).

**FIGURE 3:**
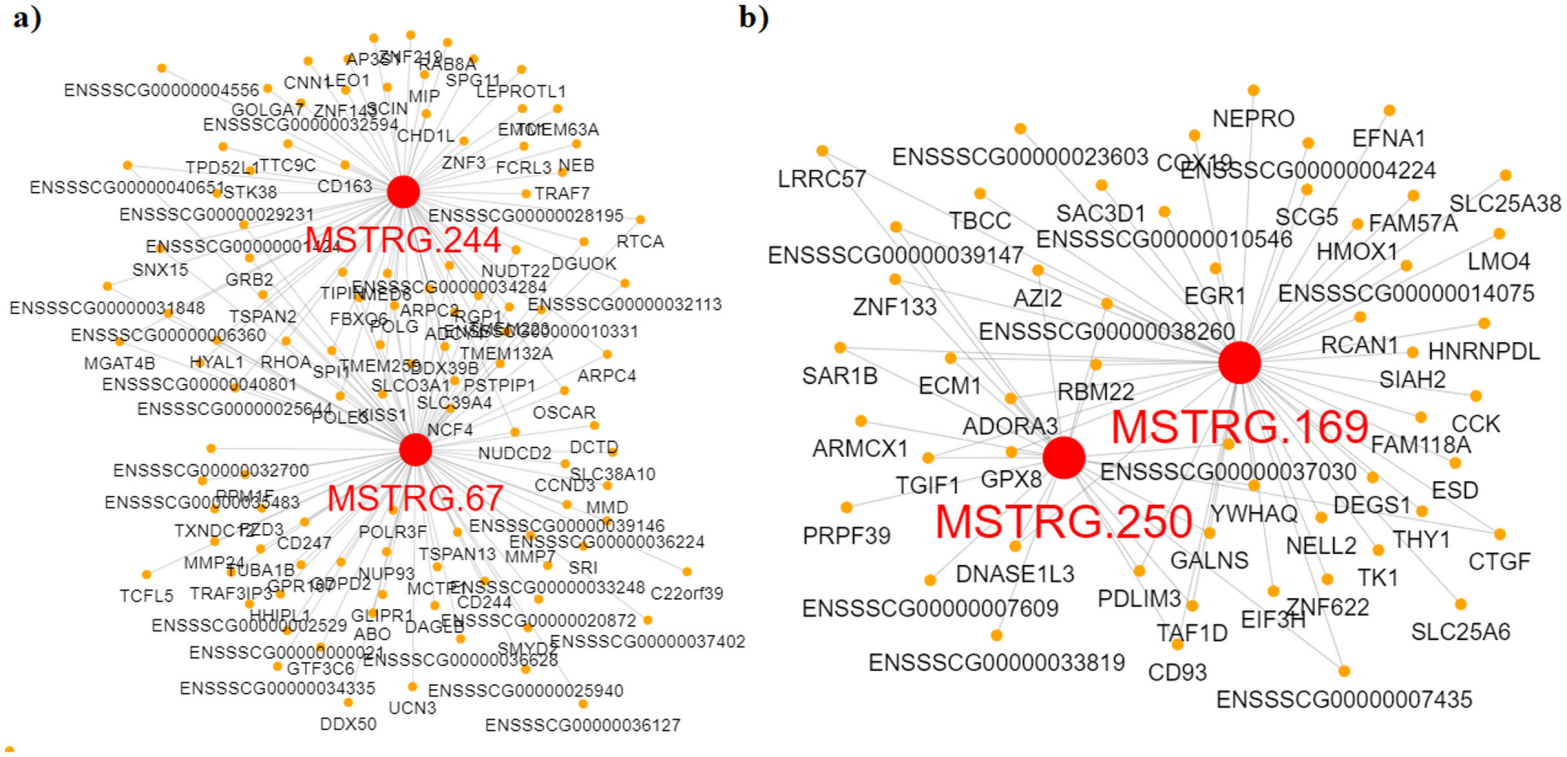
Interaction networks for the treatment containing canola oil (CO) between novel identified lncRNA and genes present in the (a) *darkslateblue* and (b) *saddlebrown* modules. The nodes in red are represented by the novel lncRNAs, while the genes are indicated in orange.

**FIGURE 4:**
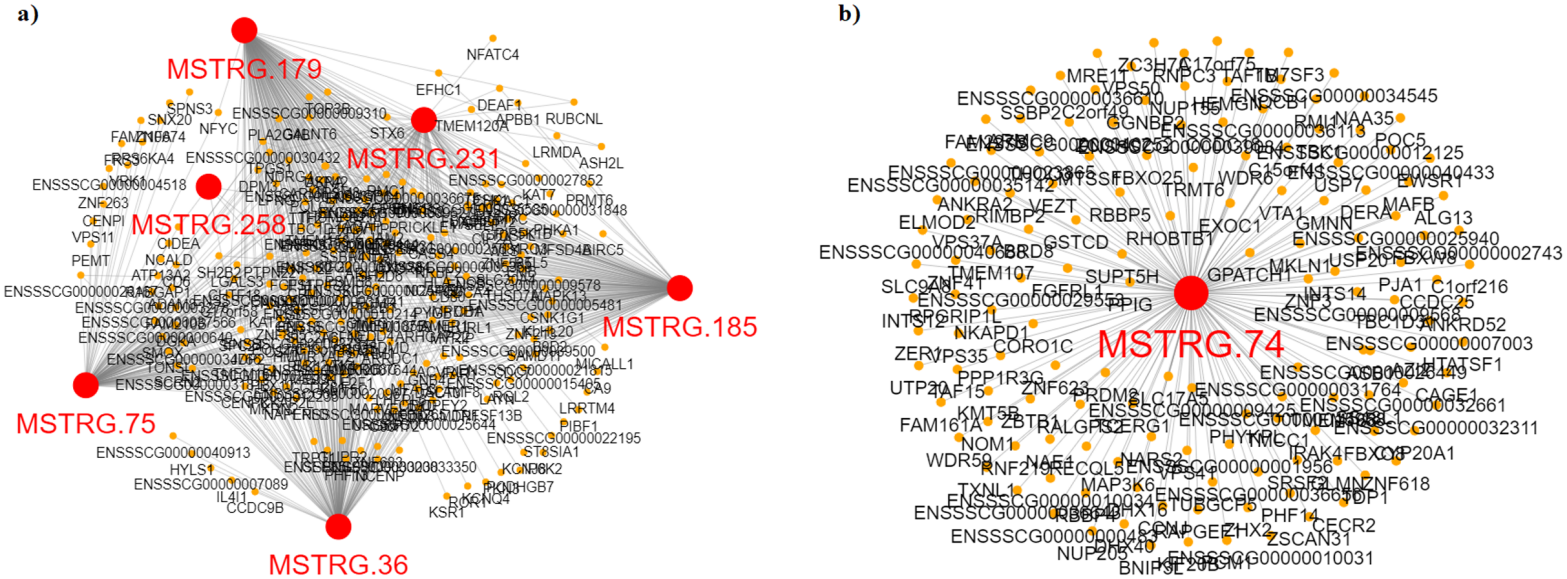
Interaction networks for the dietary treatment containing fish oil (FO) between novel identified lncRNA and genes present in the (a) *floralwhite* modules and (b) *saddlebrown*. The nodes in red are represented by the novel lncRNAs and the genes are indicated in orange.

Figures 3 and 4 show the interactions of each novel lncRNA in relation to the genes that are present in the selected unique modules and with at least one novel lncRNA in the CO and FO treatments, respectively. As can be observed from Figure 3, the lncRNAs already annotated help the novel lncRNA in gene regulation (Figure 3a), and more than one novel lncRNA acts in the regulation of the same gene (Figure 3b). The list of genes associated with the novel identified lncRNAs present in the unique modules of each treatment is available in Table S4.

### 2.4 QTLs Associated With Novel lncRNAs Identified in Unique Modules

The genes included in the unique modules identified in the CO treatment, *darkslateblue* and *saddlebrown*, overlapped with 142 and 88 annotated Quantitative Trait Loci (QTL), respectively. In addition, the *floralwhite* module, of FO, showed 989 QTLs annotated. All the novel lncRNAs present in the unique modules used for this analysis are associated with the identified QTLs as follows: *darkslateblue*: MSTRG.67 and MSTRG.244; *saddlebrown*: MSTRG.169 and MSTRG.250; and *floralwhite*: MSTRG.36, MSTRG.75, MSTRG.179, MSTRG.185, MSTRG.231, and MSTRG.258. The data are available in Table S5.

### 2.5 Enrichment Analysis of Unique Modules and QTLs

For the functional enrichment analyses, only the modules considered unique in the SOY1.5 comparisons, correlated with at least one phenotype, and with at least one novel lncRNA present were used (CO: *darkslateblue* and *saddlebrown*; FO: *floralwhite*).

For the *darkslateblue* module, in CO, 12 terms linked to cellular components were identified as enriched for the genes present in this module (Table 4), with two novel lncRNAs and three previously annotated lncRNAs. Additionally, three enriched KEGG pathways were identified (Table 5). The *saddlebrown* module had no significantly enriched terms. However, terms related to lipid and FA metabolism (*p*‐value > 0.05) were shown, such as FA metabolic process and regulation of lipid biosynthetic process. Terms considered not significant for the CO *saddlebrown* module are available in Table S6.

**TABLE 4:** Enriched GO cellular components terms identified for the *darkslateblue* module, associated with the canola oil containing treatment, after redundancy reduction analysis.

GO code	GO term	Genes	FDR
GO:0005885	*Arp2/3 protein complex*	*ARPC4, ARPC5, ARPC2*	0.002
GO:0015629	*Actin cytoskeleton*	*PSTPIP1, HCK, ARPC4, GDPD2, CNN1, SCIN, ARPC5, ARPC2, NEB, GAS2L1, RFLNA, LASP1*	0.004
GO:0035861	*Site of double‐strand break*	*CHD1L, ARPC4, ARPC5, ARPC2, UIMC1*	0.008
GO:0005764	*Lysosome*	*NCF4, GLIPR1, HLA‐DOB, SPG11, HCK, SNAP29, TMEM63A, HYAL1, CFTR, PDE1C, MMD, DAGLB, DYNC1H1, TRAF3IP3*	0.008
GO:0000323	*Lytic vacuole*	*NCF4, GLIPR1, HLA‐DOB, SPG11, HCK, SNAP29, TMEM63A, HYAL1, CFTR, PDE1C, MMD, DAGLB, DYNC1H1, TRAF3IP3*	0.008
GO:0032588	*Trans‐Golgi network membrane*	*RGP1, ST3GAL1, RAB8A, TEPSIN, MMP24*	0.01
GO:0030667	*Secretory granule membrane*	*GLIPR1, SELL, SELP, SNAP29, TMEM63A, SRI, ITGA2B, RHOA*	0.01
GO:0090734	*Site of DNA damage*	*CHD1L, ARPC4, ARPC5, ARPC2, UIMC1*	0.01
GO:0005773	*Vacuole*	*NCF4, GLIPR1, HLA‐DOB, SPG11, HCK, SNAP29, TMEM63A, HYAL1, CFTR, PDE1C, MMD, DAGLB, DYNC1H1, TRAF3IP3*	0.01
GO:0030027	*Lamellipodium*	*PSTPIP1, ACTG2, GDPD2, ARPC5, ARPC2, RHOA*	0.02
GO:0098852	*Lytic vacuole membrane*	*GLIPR1, HLA‐DOB, SPG11, SNAP29, TMEM63A, CFTR, MMD, DAGLB, TRAF3IP3*	0.03
GO:0005765	*Lysosomal membrane*	*GLIPR1, HLA‐DOB, SPG11, SNAP29, TMEM63A, CFTR, MMD, DAGLB, TRAF3IP3*	0.03

Abbreviations: FDR, significance value as a function of enrichment (FDR < 0.05); Genes, genes associated with terms; GO code, code associated with the term; GO term, term after redundancy analysis.

**TABLE 5:** Significantly enriched terms associated with the identified metabolic pathways of KEGG in the *darkslateblue* module after functional enrichment analysis for the canola oil (CO) containing treatment.

KEGG code	Pathway	Genes	FDR
KEGG:04972	*Pancreatic secretion*	*ADCY4, CA2, RAB8A, CFTR, PRKCA, RHOA*	0.02
KEGG:04666	*Fc gamma R‐mediated phagocytosis*	*HCK, ARPC4, SCIN, ARPC5, ARPC2, PRKCA*	0.02
KEGG:04530	*Tight junction*	*TUBA1B, ARPC4, RAB8A, ARPC5, ARPC2, CFTR, RHOA*	0.04

Abbreviations: Genes, genes associated with pathways; KEGG code, code associated with the road; Pathway, identified metabolic pathway; RDF, significance as a function of enrichment (FDR < 0.05).

**FIGURE 5:**
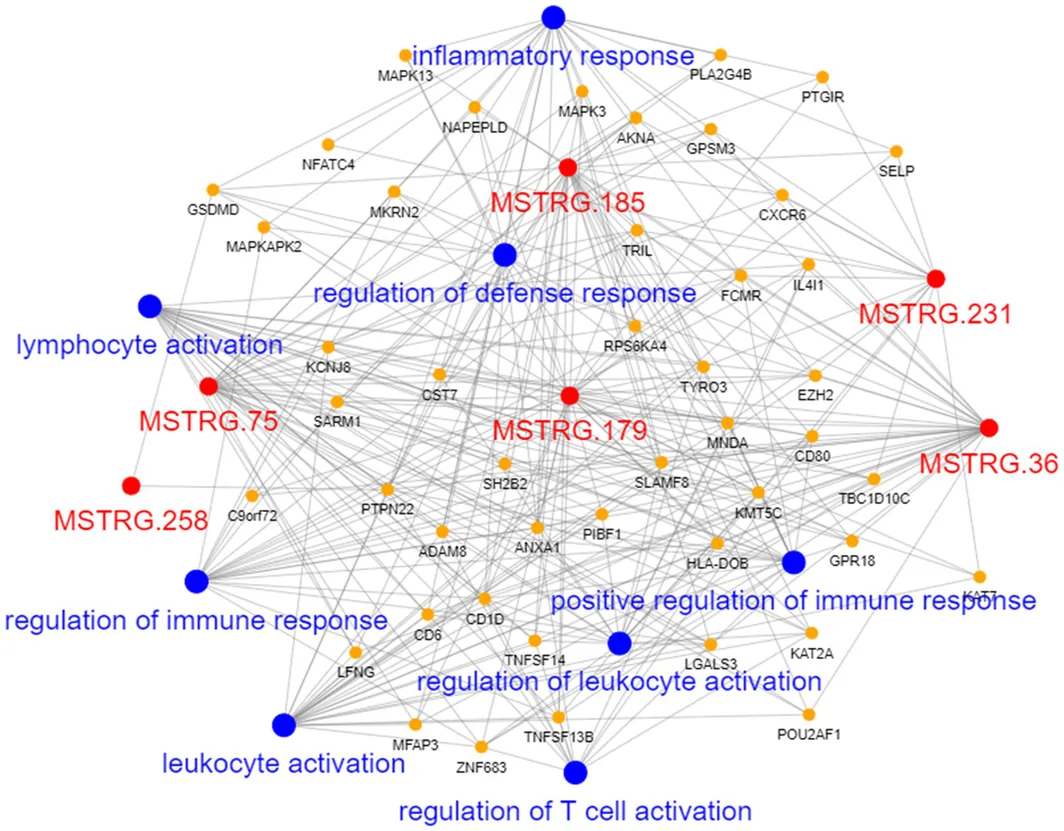
Gene interaction network among Gene Ontology (GO) terms after functional enrichment and redundancy analysis, showing novel lncRNAs identified in the *floralwhite* module under fish oil (FO) treatment. Novel lncRNAs, GO terms, and genes are shown in red, blue, and orange, respectively.

For the unique FO modules, only the *floralwhite* module was used for the functional enrichment analysis. In total, 187 terms for BP, MF, and CC GO databases, in addition to two REAC metabolic pathways, were identified. Examples of terms enriched for immune response (*p*‐value < 0.05, presence of a novel lncRNA correlated to genes associated with the terms) for the *floralwhite* modules, in FO (Table 6 and Figure 5), while the identified pathways are available in Table 7. All significant terms for the unique modules selected for the functional enrichment analysis are shown in Table S7.

**TABLE 6:** Enriched GO immune response terms identified for the *floralwhite* module, associated to the fish oil containing treatment, after redundancy reduction analysis.

GO code	GO term	Genes	*FDR*
GO:0046649	*Lymphocyte activation*	*HLA‐DOB, IL4I1, TYRO3, LGALS3, ANXA1, SLAMF8, CD1D, PTPN22, LFNG, TNFSF13B, ADAM8, CD80, TBC1D10C, CD6, POU2AF1, KAT2A, KAT7, PIBF1, TNFSF14, EZH2, GPR18, MFAP3, KMT5C, MNDA, ZNF683*	0.0001
GO:0045321	*Leukocyte activation*	*KCNJ8, HLA‐DOB, IL4I1, TYRO3, LGALS3, ANXA1, SLAMF8, CD1D, PTPN22, CST7, LFNG, TNFSF13B, ADAM8, CD80, TBC1D10C, CD6, POU2AF1, KAT2A, KAT7, PIBF1, TNFSF14, EZH2, GPR18, MFAP3, KMT5C, MNDA, ZNF683*	0.0001
GO:0002694	*Regulation of leukocyte activation*	*HLA‐DOB, IL4I1, TYRO3, LGALS3, ANXA1, SLAMF8, CD1D, PTPN22, CST7, TNFSF13B, ADAM8, CD80, TBC1D10C, CD6, KAT2A, PIBF1, TNFSF14, KMT5C, MNDA, ZNF683*	0.0001
GO:0006954	*Inflammatory response*	*KCNJ8, GPSM3, NFATC4, PLA2G4B, TYRO3, ANXA1, SELP, SLAMF8, PTPN22, GSDMD, CST7, ADAM8, CXCR6, CD6, MAPKAPK2, TRIL, NAPEPLD, AKNA, PTGIR, RPS6KA4, EZH2, MKRN2, MAPK13*	0.0006
GO:0050776	*Regulation of immune response*	*KCNJ8, HLA‐DOB, IL4I1, TYRO3, LGALS3, ANXA1, SLAMF8, CD1D, PTPN22, SH2B2, TNFSF13B, ADAM8, C9orf72, CD80, MAPKAPK2, FCMR, TRIL, MKRN2, SARM1, KMT5C, MNDA, ZNF683*	0.002
GO:0050863	*Regulation of T cell activation*	*HLA‐DOB, IL4I1, LGALS3, ANXA1, CD1D, PTPN22, TNFSF13B, ADAM8, CD80, CD6, KAT2A, TNFSF14, ZNF683*	0.004
GO:0031347	*Regulation of defense response*	*KCNJ8, GPSM3, TYRO3, ANXA1, SLAMF8, CD1D, PTPN22, GSDMD, CST7, ADAM8, MAPKAPK2, TRIL, NAPEPLD, AKNA, MKRN2, SARM1, MNDA, MAPK13, MAPK3*	0.006
GO:0050778	*Positive regulation of immune response*	*KCNJ8, HLA‐DOB, TYRO3, LGALS3, ANXA1, CD1D, PTPN22, SH2B2, TNFSF13B, ADAM8, CD80, MAPKAPK2, FCMR, TRIL, SARM1, KMT5C, MNDA, ZNF683*	0.007

Abbreviations: Genes, shows the genes associated with pathways; RDF, significance as a function of enrichment (FDR < 0.05); REAC code, code associated with the road; Route, the identified route is listed.

**TABLE 7:** KEGG metabolic pathways identified for genes present in the *floralwhite* module after functional enrichment analysis for the fish oil treatment.

REAC code	Pathway	Genes	FDR
REAC:R‐HSA‐141424	Amplification of signal from the kinetochores	*B9D2, CENPI, INCENP, CENPK, PLK1, SPC24, BIRC5*	0.04
REAC:R‐HSA‐141444	Amplification of signal from unattached kinetochores via a MAD2 inhibitory signal	*B9D2, CENPI, INCENP, CENPK, PLK1, SPC24, BIRC5*	0.04

Abbreviations: Genes, shows the genes associated with pathways; RDF, significance as a function of enrichment (FDR < 0.05); REAC code, code associated with the road; Route, the identified route is listed.

The enrichment analysis of previously identified QTLs for the modules containing at least one novel lncRNA pointed to 21, 10, and 46 enriched QTLs for the *darkslateblue*, *saddlebrown*, and *floralwhite* modules, respectively, where all modules were enriched for QTLs influencing the *Health*, *Meat and Carcass*, *Reproduction*, *Exterior*, and *Production* type categories considered in the AnimalQTLdb. Information on QTL enrichment results can be found in Table 8, while the general list of QTLs (significant and non‐significant) identified after enrichment analysis is available in Table S8.

**TABLE 8:** Enriched quantitative trait loci (QTLs) (FDR < 0.05) for each unique module containing at least one novel lncRNA identified in relation to the canola and fish oil treatments.

Treatment	Module	FDR	QTL	QTL type	QTLs co‐localized	QTLs annotated
CO	*saddlebrown*	7.5 × 10^−8^	*Mean corpuscular hemoglobin content*	Health	6	23
CO	*saddlebrown*	1.5 × 10^−5^	*Teat number*	Reproduction	7	23
CO	*darkslateblue*	0.0003	*Teat number*	Reproduction	7	39
CO	*darkslateblue*	0.003	*Feed conversion ratio*	Production	4	39
CO	*darkslateblue*	0.004	*Thoracolumbar vertebra number*	Exterior	2	39
CO	*darkslateblue*	0.006	*Red cell distribution width*	Health	2	39
CO	*darkslateblue*	0.008	*Body weight*	Production	3	39
CO	*darkslateblue*	0.023	*Lean meat percentage*	Meat and Carcass	2	39
CO	*darkslateblue*	0.039	*Intramuscular fat content*	Meat and Carcass	3	39
CO	*darkslateblue*	0.049	*Coping behavior*	Exterior	3	39
FO	*floralwhite*	1.6 × 10^−155^	*Coping behavior*	Exterior	133	272
FO	*floralwhite*	3.7 × 10^−18^	*CD3‐negative, CD8‐negative leukocyte percentage*	Health	34	272
FO	*Saddlebrown*	*3.74 × 10* ^*−19*^	*Teat number*	Reproduction	7	10
FO	*floralwhite*	5.7 × 10^−6^	*Linolenic acid content*	Meat and Carcass	6	272
FO	*floralwhite*	0.002	*Marbling*	Meat and Carcass	5	272
FO	*floralwhite*	0.003	*Loin weight*	Meat and Carcass	4	272
FO	*floralwhite*	0.005	*Neutrophil count*	Health	5	272
FO	*floralwhite*	0.008	*Time spent in feeder*	Production	3	272
FO	*floralwhite*	0.037	*Enterotoxigenic E* *. coli* *susceptibility*	Health	4	272
FO	*floralwhite*	0.039	*Litter weight, total*	Reproduction	3	272

Abbreviations: FDR, False Discovery Rate indicating the significance of QTL enrichment; Module, Unique module associated with the treatment; QTL type, classification of the QTL; QTL, Identifier of the detected Quantitative Trait Locus; QTLs annotated, Number of QTLs annotated in the Animal QTL Database; QTLs co‐localized, Number of co‐localized QTLs; Treatment, Dietary treatment applied.

## 3 Discussion

In this study, we aimed to identify novel lncRNAs from different sources of FAs using co‐expression networks to evaluate how different diets affect regulatory elements of the liver. The development of strategies to improve human health can be achieved through nutrigenomics (Afman and Müller 2012), contributing to understanding how different diets influence the emergence and progression of diseases through the modulation of gene expression (Dongiovanni and Valenti 2017).

A limitation was that the RNA‐seq libraries were prepared using a poly(A)‐enrichment strategy. Consequently, the identified lncRNA landscape represents only the polyadenylated fraction of the transcriptome, and non‐polyadenylated lncRNAs were likely underrepresented or not detected. Long‐term dietary patterns have been shown to affect disease development through regulatory molecules such as lncRNAs (Lei et al. 2022; Shabgah et al. 2021).

Overall, lncRNAs can play a regulatory role in gene expression networks through interactions with proteins, chromatin, and other RNA classes (Rinn and Chang 2012), making them potential prognostic biomarkers for diseases such as nonalcoholic fatty liver disease (NAFLD) and diabetes mellitus (Alharbi 2024; Jathar et al. 2017; Zeng et al. 2023).

Fatty acids derived from diets rich in DHA and EPA, present in fish oil and classified as n‐3 FAs, can inhibit inflammation and improve insulin sensitivity, suggesting protective effects against metabolic disorders (Oh et al. 2010). In contrast, MUFAs, which are abundant in canola oil, modulate immune cell formation and trafficking in autoimmune and metabolic conditions (Lemus‐Conejo et al. 2021; Raeisi‐Dehkordi et al. 2021). The influence of these dietary FAs on gene expression likely occurs through regulatory molecules such as lncRNAs, reinforcing their role as mediators between dietary composition and disease‐related transcriptional change (Afman and Müller 2012; Dongiovanni and Valenti 2017; Lei et al. 2022; Shabgah et al. 2021).

Distinct co‐expression modules were identified for the two treatments compared to the control (SOY1.5): *darkslateblue* and *saddlebrown* (CO treatment), and *floralwhite* (FO treatment). These modules correlated with phenotypes such as ether extract muscle (EEM), SFA, MUFA, IL‐6, IL‐18, and TNF‐α, and contained novel lncRNAs correlated with their respective genes, indicating that dietary FAs modulate immune and metabolic traits through lncRNA‐gene interactions.

The *darkslateblue* module, associated with the CO treatment, showed enrichment for cellular components such as the actin cytoskeleton and lysosomal membrane, and included genes involved in cytokine signaling. Among the genes in this module, the diacylglycerol lipase beta (*DAGLB*) gene is particularly notable. Different studies aimed at understanding the role of the DAGL family in metabolic functions and immune signaling have shown that *DAGLB* regulates the TNF‐α response stimulated by lipopolysaccharides, while attenuating dendritic‐cell inflammatory signaling, where modulation preserves essential adaptive immune functions and highlights the potential of targeting lipid pathways for immunomodulation (Hsu et al. 2012; Shin et al. 2019).

Metabolic pathways were enriched in the *darkslateblue* module, such as pancreatic secretion, where one of its associated genes is *RAB8A*, a gene that can prevent the generation of hyperinflammation events by decreasing pro‐inflammatory cytokines and increasing anti‐inflammatory cytokines (Luo et al. 2014). The role of *RAB8A* as a regulator of cytokine secretion, including IL‐6, has been described, and palmitic acid, present in SFA‐rich diets, has been reported to increase IL‐6 release when cells are stimulated by lipopolysaccharides (Luo et al. 2014).

The effect may be associated with the activation of the toll‐like receptor 4 through SFA (Lee et al. 2001). These findings align with the correlation results obtained in our study, where the genes present in the *darkslateblue* module were correlated with the SFA, MUFA, IL‐6, and TNF‐α traits.

The *saddlebrown* module, also specific to the CO treatment, included genes associated with lipid transport and insulin resistance, notably Secretion Associated Ras Related GTPase 1B (*SAR1B*) and Delta 4‐Desaturase, Sphingolipid 1 (*DEGS1*), both correlated with novel lncRNAs (MSTRG.169 and MSTRG.220). Mutations in *SAR1B* have been reported to impair chylomicron (CM) trafficking (Auclair et al. 2023), which is responsible for transporting medium‐ and long‐chain FAs between the endoplasmic reticulum and the Golgi complex (Tomkin and Owens 2012). Impaired trafficking reduces CM secretion and leads to deficiencies in essential vitamins and FAs (Schlöndorff and Blobel 1999). In transgenic mice fed high‐fat diets, Levy et al. (2014) showed that SAR1B mutations were associated with increased body weight, lipid accumulation, insulin resistance, and hepatic steatosis.

Additionally, the *DEGS1* gene has recently been described as a possible target gene to combat obesity related to insulin resistance (Bikman et al. 2012). According to Barbarroja et al. (2015), their data provide evidence that selectively manipulating *DEGS1* in white adipose tissue can improve adipose tissue homeostasis and metabolic complications associated with obesity.

Research has shown that lncRNAs mediate biological processes in cells, including glucose and lipid metabolism (Muret et al. 2019; Zhang et al. 2020). This finding supports the work of Zhang et al. (2020), who discussed the involvement of lncRNAs in the pathogenesis of Type 2 Diabetes (T2D). According to their study, lncRNAs could serve as potential biomarkers or therapeutic targets for treating T2D. Furthermore, understanding the role of lncRNAs in metabolic regulation, particularly in relation to obesity, could provide new avenues for medical intervention and help prevent the development of related disorders. This is relevant given that lncRNAs are implicated in obesity‐related syndromes characterized by altered lipid, cholesterol, and glucose metabolism (Lu et al. 2021).

Together, these findings highlight that the lncRNAs correlated with *SAR1B* and *DEGS1* may regulate lipid metabolism and energy balance in response to dietary fatty acids, reinforcing the link between FA source, lipid transport, and insulin sensitivity.

The *floralwhite* module, which was unique to the FO treatment, included several genes and novel lncRNAs associated with both metabolic and immune functions. Recently, it was proposed that the Fibroblast Growth Factor 11 (*FGF11*) gene, identified as a possible target in cis for lncRNA in the *floralwhite* module in our analyses, is of great importance due to its role in adipocyte differentiation, and may also contribute to metabolic diseases, where Peroxisome Proliferator‐Activated Receptor γ (*PPAR‐γ*) expression helped in the effect of *FGF11* during adipogenesis (Lee et al. 2019). Cho et al. (2022), during their research, found that the *FGF11* gene is a possible target for the treatment of obesity. Our results indicate that one of the novel lncRNAs identified in the *floralwhite* module of FO, MSTRG.75, is possibly acting in the regulation of *FGF11*, and together with synthetic PPAR‐γ drugs, they may become one of the alternatives to improve insulin sensitivity, acting in the modulation of lipid and glucose metabolism (Lehmann et al. 1995).

The correlation results observed for the *floralwhite* module align with the QTL enrichment analysis, supporting the functional relevance of the novel lncRNAs identified. This module showed both significant and non‐significant enrichment for QTLs related to LA content (*p*‐value < 0.05) and intramuscular fat (*p*‐value > 0.05), respectively. Notably, this module also showed positive correlations with MUFA and SFA, which may reflect involvement in FA‐related pathways. These findings suggest a potential regulatory role of the novel lncRNAs, possibly through interaction with *FGF11*, in muscle FA composition. Furthermore, based on this evidence, the additional genes within the unique modules may represent novel trans targets influenced by these lncRNAs.

During the term enrichment analysis for genes in the *floralwhite* module of the FO treatment, one gene stood out due to its presence in most terms and its correlation with several novel lncRNAs identified in the module: Protein Tyrosine Phosphatase Non‐Receptor Type 22 (*PTPN22*). This gene encodes a tyrosine phosphatase (Cloutier and Veillette 1999) that regulates pathways in various cell types, such as T‐cell (Hasegawa et al. 2004) and B‐cell (Dai et al. 2013) signaling.

*PTPN22* was correlated with SFA, MUFA, IL‐18, and with most novel lncRNAs identified in the *floralwhite* module (MSTRG.36, MSTRG.75, MSTRG.179, MSTRG.185, and MSTRG.258). Several studies suggest that *PTPN22* plays a key role in modulating immune responses, particularly through its regulation of myeloid cells such as macrophages and dendritic cells (Bottini and Peterson 2014; Chang et al. 2013; Fousteri et al. 2013), contributing to inflammation via the secretion of cytokines such as IL‐18, IL‐6, and TNF‐α (Kawai and Akira 2011). Mutations in this gene impair T‐cell responsiveness and increase susceptibility to autoimmunity (Sanchez‐Blanco et al. 2018), which can lead to Type 1 diabetes (T1D) (Giza et al. 2013). T1D involves immune‐mediated destruction of pancreatic β‐cells responsible for insulin production (Theofilopoulos and Bona 2002), driven by increased levels of pro‐inflammatory cytokines and activated T and B cells (Naser et al. 2013). These findings are consistent with Niinistö et al. (2017), who reported that FA intake may contribute to T1D development, while consumption of fish‐derived FAs may reduce the risk during childhood.

This agrees with the results of our enrichment analysis, which revealed several significant terms related to pancreatic alterations, including B‐cell activation (*p*‐value = 0.03) and regulation of B‐cell activation (*p*‐value = 0.03).

Other genes are related to the novel lncRNAs detected in the present work, including Myeloid Cell Nuclear Differentiation Antigen (*MNDA*) and Annexin A1 (*ANXA1*). According to Jin et al. (2013), *MNDA* is strongly linked to T1D, although its functions and more studies are needed to verify the relation to the disease, while *ANXA1* is thought to be linked to glucose and FA metabolism (Grewal et al. 2019).

The study by Purvis et al. (2019), observed that plasma levels of *ANXA1* were associated with liver fat content in patients with T2D, pointing to a possible relationship between this gene and lipid metabolism. This information is in agreement with the results obtained in the present work, where both genes were correlated with phenotypes related to FA and associated with novel lncRNAs, showing how the use of dietary FA of animal origin, especially fish, can affect the expression profiles of genes involved in the immune system.

The *ADAM8* gene (ADAM Metallopeptidase Domain 8), from the family of disintegrin and metalloprotease (*ADAM*) (Schlöndorff and Blobel 1999), found in the *floralwhite* module of the FO diet, is related to the development of obesity, contributing to insulin resistance and promoting adipose tissue inflammation (Xu et al. 2003). This module was also correlated with IL‐18 (0.64), a pro‐inflammatory cytokine associated with obesity (Bruun et al. 2007). IL‐18 downregulation is considered a good indicator of improved metabolic status when replacing SFA with n‐3 FAs, making IL‐18 a potential biomarker connecting dietary FA quality and insulin resistance (Sergi et al. 2023), Additionally, elevated production of IL‐18 and IFN‐γ is characteristic of several human autoimmune diseases, including T1D (Dinarello et al. 2013).

## 4 Conclusion

The results obtained here indicate a potential regulatory role of lncRNAs on genes involved in inflammatory responses, showing a differential pattern depending on the type of dietary fatty acids consumed. Both SFA and MUFA appear differently to modulate these lncRNAs. This regulation may influence immune responses, potentially preventing or promoting disease development according to the levels of fatty acids consumed. These findings highlight lncRNAs as potential biomarkers for the development of diagnostic methods and therapeutic strategies for human diseases.

Genes significantly expressed in response to canola oil treatment were not directly associated with autoimmune diseases, but the modules containing these genes were correlated with cytokines (TNF‐α and IL‐6). All the genes mentioned were correlated with at least one novel lncRNA identified in the respective modules, many of which are associated with inflammatory processes and autoimmune diseases. *PTPN22*, *MNDA*, *ANXA1*, *ADAM8*, *FGF11*, *SAR1B*, *DEGS1*, *RAB8A*, and *DAGLB* are possible candidate genes for studies of metabolic diseases such as obesity and diabetes.

Further studies are needed to define the regulatory role of these novel lncRNAs over their target genes and to understand how this regulation affects biological processes associated with inflammatory responses and metabolic diseases. In particular, experimental validation through in vitro and *in silico* approaches will be essential to confirm the impact of these lncRNAs on their targets and their potential as modulators of immune and metabolic phenotypes.

## 5 Methods

### 5.1 Ethics Statement

All procedures involving animals were evaluated and approved by the Ethics Committee for the Use of Animals (CEUA) of the “Luiz de Queiroz” College of Agriculture (ESALQ/USP), receiving protocol number 2018.5.1787.11.6 and CEUA number 2018‐28.

### 5.2 Animals and Experimental Diets

Seventy‐two immunocastrated Large White male pigs that were homozygous negative (NN) for the halothane mutation (*RYR1* gene) were used in this 98‐day experiment. Pigs aged 71 ± 1.8 days were blocked by initial body weight of 28.44 ± 2.95 kg. All experimental procedures for this experiment have been described in detail by Almeida et al. (2021) and Fanalli, da Silva, Gomes, et al. (2022). Briefly, pigs were randomly assigned to one of four dietary treatments, with six replicate pens per treatment and three pigs per pen, totaling 18 pigs per treatment. Pigs were fed in a six‐phase feeding program: grower I—Days 0–21; grower II—Days 21–42; finisher I—Days 42–56; finisher II—Days 56–63; finisher III—Days 63–70; and finisher IV—Days 70–98. Pigs had ad libitum access to feed and water throughout the experiment. Diets were fed in mash form.

Dietary treatments consisted of corn‐soybean meal growing‐finishing diets supplemented with 1.5% soybean oil (SOY1.5) or 3% oil from either soybean oil (SOY3.0), canola oil (CO), or fish oil (FO). All diets were formulated to meet or exceed the Rostagno et al. (2011) (Santiago et al. 2011) nutrient requirements. At the completion of the study, Day 98, all pigs (*n* = 72) were slaughtered with a final body weight of 133.9 ± 9.4 kg. Pigs were processed according to standard industry procedures, and liver samples were collected immediately post exsanguination. Samples were frozen in liquid nitrogen and stored at −80°C until analysis.

### 5.3 Fatty Acids and Cytokine Profiles

The composition of FA was previously investigated by our research group and described in Almeida et al. (2021). The FA profile was performed by Bligh and Dyer and methylated according to the procedure outlined by AOCS (Method AM 5‐04).

Blood was sampled from the jugular vein of 27 pigs 4 days before slaughter and immediately transferred into non‐anticoagulant vacuum tubes for subsequent analyses (Becton Dickinson Vacutainer Systems, Franklin Lakes, NJ, USA). The samples were then stored at room temperature for 2 h, after which they were centrifuged at 3000 × g for 10 min to obtain serum. The serum was stored in duplicate 1.5‐mL tubes at −80°C (Fanalli, da Silva, Gomes, et al. 2022; Fanalli, da Silva, Petry, et al. 2022). The proteins were extracted from the right lobe of the liver. The protein concentration was then determined using the Pierce BCA Protein Assay Kit, with absorbance read at 520 nm. A multiplex sandwich ELISA assay was performed using a customized Milliplex Map Kit (Merck), following the manufacturer's instructions, for determination of interleukin (IL)‐10, IL‐1‐beta, IL‐6, IL‐18, interferon (IFN)‐gamma, and tumor necrosis factor (TNF)‐alpha (Ciconello et al. 2025).

### 5.4 RNA Extraction, Library Preparation, and Sequencing

Total RNA was extracted from liver samples using the RNeasy Mini Kit (QIAGEN, Hilden, Germany) following the manufacturer's guidelines. The verification of the total RNA quality was determined using a NanoDrop 2000 spectrophotometer (Thermo Fisher Scientific), and the integrity was evaluated with an Agilent 2100 Bioanalyzer (Agilent, Santa Clara, CA, USA). After verifying the quality and integrity of the samples, a total of 2 μg of total RNA from each sample was used for the preparation of the library according to the protocol described in the TruSeq RNA Sample Preparation kit v2 (Illumina, San Diego, CA).

The average size of the libraries was estimated using the Agilent Bioanalyzer 2100 (Agilent, Santa Clara, CA, USA) and quantified using quantitative PCR with the KAPA library quantification kit (KAPA Biosystems, Foster City, CA, USA). Then, samples were diluted and pooled (three pools of six samples each). Five lanes with 36 samples pooled with different barcodes were used in two sequencing flow cells, using the TruSeq PE Cluster kit v4‐cBot‐HS (Illumina, San Diego, CA, USA). Sequencing was performed on the HiSeq2500 equipment (Illumina, San Diego, CA, USA) with the TruSeq SBS v4‐HS kit (200 cycles), following the manufacturer's instructions.

### 5.5 Quality Control and Alignment

The quality of the RNA‐Seq readings was verified with the FastQC (v.0.11.8) software (Andrews 2010), before and after trimming. Subsequently, parameters such as Phread = 33 and base pair length greater than 70 bp were used after cutting the sequences by quality and eliminating adapters with the Trim Galore (v.0.6.5) software (Krueger 2015).

Alignment and mapping were performed using STAR (v.2.7.9a) *software* (Dobin et al. 2013) using the reference *Sus Scrofa 11.1* (http://www.ensembl.org/Sus_scrofa/Info/Index) available in the Ensembl Genome Browser version 112, via coordinates (option ‐‐*sorted by coordinate*—genomic localization). The input file for this analysis must contain the *spliced RNA‐seq read alignments*, indicating the genomic strand from which the RNA generates the original read. For this, the option *‐‐outSAMstrandField intronMotif* was used. Alignments containing non‐canonical joins were also filtered and removed through the *‐‐outFilterIntronMotifs RemoveNoncanonical*.

### 5.6 Identification of Novel lncRNAs and Quantification of Transcripts

The assembly of transcripts was done using the StringTie (v.2.2.1) software (Pertea et al. 2015) for all samples. The merging of the results from each sample into a single file was performed using filters with the *‐‐merge* option, where transcripts with a length greater than 200 bp and a minimum of 0.1 TPM were retained. Subsequently, the transcripts were compared and classified using the GffCompare (v.0.12.6) software (Pertea and Pertea 2020), adding Class Codes. The following classes were selected to continue the filtering stage for potential novel lncRNA: “i” (intronic long noncoding RNA), “j” and “o” (long noncoding sense transcript), “u” (long intergenic noncoding RNA), and “x” (long noncoding antisense transcript).

The GffRead (v.0.12.7) software (Pertea and Pertea 2020) was then used, obtaining the genomic sequence of the selected transcripts, thus making it possible to perform the identification and removal of ORF (Open Reading Frames) using the Orfipy (v.0.0.4) software (Singh and Wurtele 2021). For this analysis, a minimum value of 300 bp between start and stop codons was established as a parameter for its identification, being removed by the faSomeRecords.py tool (https://github.com/santiagosnchez/faSomeRecords) later. The generated fasta output file was used to find potential protein‐coding sequences using the Coding Potential Calculator 2 (CPC2, v.1.0.1) software (Kang et al. 2017), where they were classified as “noncoding” and “coding”. Transcripts classified as coding were removed by the faSomeRecords.py tool.

The transcripts were tested for similarity with the UniProt/SwissProt database using the Basic Local Alignment Search Tool (BLAST+, v.2.14.0) software (Camacho et al. 2009) and applying the BLASTX option. Transcripts containing homologues were removed from the database (E‐value < 10^−6^).

Data from the lncRNA‐associated transcripts already annotated in the *Sus scrofa* 11.1 reference genome (*n* = 2930) were included in the gtf file generated by StringTie during the identification of novel lncRNA. This process was also carried out by the *‐‐merge* option of the StringTie software. Hence, both annotated and novel lncRNAs were incorporated into the final dataset.

The RNA‐Seq by Expectation Maximization (RSEM, v.1.3.3) software (Li and Dewey 2011) was used to estimate the gene expression of the transcripts present in the gtf file generated at the end of the identification of novel lncRNA. It is important to note that in the stage of quantification of the transcripts by RSEM, the gtf file contains the lncRNA, including novel lncRNA and lncRNA previously annotated in the reference genome, and other transcripts (mRNA and other classes).

### 5.7 Analysis of Co‐Expression Networks, Correlation With Phenotypes of Interest, and Identification of QTL Associated With Novel lncRNAs

Genes with FPKM values > 0.2 for at least 50% of the samples were kept for the subsequent analyses. Only genes shared between the four treatments were selected for the co‐expression analysis. The R CoExpNets package (Botía et al. 2017; Langfelder and Horvath 2008) was used to construct the co‐expression networks. In this step, co‐expressed gene modules were identified using a minimum of 30 genes per module. In addition, a total of 20 interactions were defined for the k‐means algorithm used to refine the clustering of genes in each module. Genes not clustered to any module were assigned to the grey60 module. Both the minimum module size and the minimum exchanged genes parameters used in this analysis correspond to the default settings implemented in the CoExpNets R package.

The reference treatment (SOY1.5) was used as a reference for comparisons between sets of modules. In this way, all modules identified in SOY3.0, FO, and CO were compared against SOY1.5. Through Fisher's exact tests applied to each pair of modules between SOY1.5 and one of the treatments, it was possible to detect if the reference module had a counterpart of any of the modules detected in each treatment. The presence of a counterpart in the reference module set was defined from a significant overlap between two modules, where the threshold for the *p*‐value was set to 0.05 after the Bonferroni correction for multiple tests. Modules without any counterpart among the modules of the reference were defined as unique for the treatment in question, while the others were classified as shared.

The correlation analysis between the Eigengene Modules (EM) and phenotypic characteristics for Fat Thickness (FT), Ether Extract of Liver (EEL), and Muscle (EEM), Saturated Fatty Acids (SFA), Monounsaturated (MUFA), and Polyunsaturated (PUFA) and Omega 6:3 Ratio (n6:n3 ratio) was performed through the R Weighted Correlation Network Analysis (WGCNA) package (Langfelder and Horvath 2008). The phenotypes with non‐normal distribution, defined by the Shapiro–Wilk test (p‐value < 0.05), were correlated with the modules by the Spearman method (FT, EEL, and EEM), while for the phenotypes considered with a normal distribution (p‐value > 0.05), the Pearson method (SFA, MUFA, PUFA, and n6:n3 ratio) was used.

Immunity‐related phenotypes, such as Interleukin 10 (IL‐10), Interferon‐gamma (IFN‐γ), Interleukin 1‐beta (IL1‐β), Interleukin 6 (IL‐6), Interleukin 18 (IL‐18), and Tumor necrosis factor‐alpha (TNF‐α), were also tested for normality by the Shapiro–Wilk test and tested for correlation with the ME values of the modules identified in each treatment. The Spearman method was used for phenotypes considered non‐normal (IL‐10, IFN‐γ, and TNF‐α) and Pearson for phenotypes considered normal (IL1‐β, IL‐6, and IL‐18).

The phenotypes were adjusted by a simple linear model in R, with a vector of means of the phenotypic values, with block and parent being the fixed effects. Thus, the adjustment of the phenotypes will be obtained by the Equation (_1_):

(1) $${\text{𝑦}}_{\mathrm{ijk}}=\text{𝜇}+D_{i}+b_{j}\;{\text{+𝜀}}_{\mathrm{ijk}}$$

where **𝐲**_ijk_ is the adjusted/corrected phenotype, 𝜇 the general mean vector for the phenotypes; **D** and **b** are the fixed effects of diet and block; and **ε** is the residual vector.

The unique modules that presented at least one novel lncRNA and a correlation with any of the phenotypes studied were selected for the analysis of functional enrichment of unique modules (p‐value < 0.05) and QTLs.

### 5.8 Functional Enrichment of Unique Modules and Identified QTLs

The unique modules (without counterparts in relation to the modules of the reference treatment) significantly correlated with at least one phenotype and containing at least one novel lncRNA were subjected to a functional enrichment analysis using different databases. The R gprofiler2 package (Kolberg et al. 2020) was used to perform the enrichment analysis from Gene Ontology (GO), Biological Process (BP), Molecular Function (MF), and Cellular Components (CC) terms and metabolic pathways from Kyoto Encyclopedia of Genes and Genomes (KEGG) and Reactome (REAC) using the human ( *Homo sapiens* ) database as the reference due to the more comprehensive functional annotation available for humans compared with *Sus scrofa* , whose transcriptome remains less well characterized across different tissues and organs (Tang et al. 2017).

The GO terms and metabolic pathways were significantly enriched after the application of the correction for multiple tests of False Discovery Rate (FDR) (Benjamini and Hochberg 1995) of 5%. Then, a reduction analysis of redundant terms for GO terms was performed by the R rrvgo package (Sayols 2023), where similar terms based on their semantic similarity were grouped through the Wang method, and the threshold value of 0.7 was applied for the redundancy reduction.

Based on chromosomal coordinates of the novel lncRNAs present in each unique module and of QTLs (Quantitative Trait Loci) annotated in the Animal QTLdb database (Hu et al. 2019), the R package GALLO (Fonseca et al. 2020) was used to identify co‐localization of lncRNAs and QTLs within distances of up to 100 kb upstream and downstream. For the analysis of QTL enrichment, the correction for multiple FDR tests was also applied, in which only the QTLs significantly enriched and that presented a number greater than two QTLs were used for the discussion.

### 5.9 Analysis of Potential Target Genes in Cis‐Associated With Novel lncRNAs

For the identification of target genes in cis of the novel lncRNA, the R GALLO package (Fonseca et al. 2020) was also used. For that, genes mapped on the same chromosome at a maximum distance of 50 kb in relation to the start and end coordinates of the novel lncRNA were used. After identifying the possible targets in cis, the RNAplex software v.2.6.4 (Tafer and Hofacker 2008) was used to define the significance between the sequence interactions of the novel lncRNA and their potential targets in cis, where the energy value established was *E* < 30.

### 5.10 Interaction Networks Between Novel lncRNAs and Genes in Meaningful, Unique Modules

The R igraph (Csardi 2014) and VisNetwork (Almende B.V. and Contributors and Thieurmel 2015) packages were used to graphically display the relationship between the lncRNA and the potential target genes present in the co‐expression networks. An adjacency threshold > 0.02 was used in order to cover the largest possible number of genes correlated by novel lncRNA in the network.

## Author Contributions

L.E.N., B.P.M.S., P.A.S.F., A.S.M.C., and B.G.‐G. contributed to the development of this study. B.P.M.S., M.C.D., C.S.O., F.N.C., S.L.F., F.A.O.F., I.C.G., V.V.A., and A.S.M.C. supported with the revision and suggestions for the writing of this manuscript. A.S.M.C. obtained financial support. All authors read and approved the final manuscript.

## Funding

This study was supported by the São Paulo State Research Foundation (FAPESP, Grant numbers: 2017/25180‐2 to A.S.M. Cesar, 2023/06442‐7 and 2023/14662‐7 to L.E. Nascimento, 2021/10394‐2 to F.N. Ciconello, 2022/08142‐8 to M.C. Durval, 2022/10780‐2 to B.P.M. da Silva and S.L. Fanalli to 2022/10643‐5). This study was financed in part by the Coordenação de Aperfeiçoamento de Pessoal de Nível Superior—Brazil (CAPES)—Finance Code 88887.705134/2022‐00 to L.E. Nascimento. The Brazilian National Council for Scientific and Technological Development (CNPq, 303165/2022‐7) provided a research fellowship to A.S.M.C.

## Consent

The authors have nothing to report.

## Conflicts of Interest

The authors declare no conflicts of interest.

## Supporting information


**Data S1:** Total RNA extraction—Liver—NanoDrop‐generated concentration data and RNA Integrity Number (RIN).

**Data S2:** Information regarding novel lncRNAs identified in the liver of pigs fed different supplemental diets.

**Table S1:** Unique genes for each type of comparison between treatments and their classifications.

**Table S2:** Details about the genes and classifications for each type of treatment used in the study (Canola, Fish and, Soybean Oil).

**Table S3:** Comparisons between the genes and phenotypes used during WGCNA analysis.

**Table S4:** List of genes associated with the novel identified lncRNAs present in the unique modules of each treatment.

**Table S5:** QTLs co‐localized through the genomic coordinates of the novel lncRNAs identified.

**Table S6:** Enrichment for the terms of the Saddlebrown module, for treatment using Canola Oil 3% 3%.

**Table S7:** GO Terms related to the modules identified and selected according to the selection criteria for the functional enrichment analysis.

**Table S8:** QTL enrichment for modules containing putative lncRNAs according to their genomic coordinates and quality filters.

## Data Availability

The dataset used in this study is available in the European Nucleotide Archive (ENA) repository (EMBL‐EBI), under accession PRJEB50513 (https://www.ebi.ac.uk/ena/browser/view/PRJEB50513).
